# Small bowel obstruction from migrated intragastric balloon

**DOI:** 10.1016/j.amsu.2018.09.031

**Published:** 2018-09-27

**Authors:** Ayad Ahmad Mohammed, Sardar Hasan Arif, Abdulwahid M. Salih, Fahmi Hussein Kakamad

**Affiliations:** a University of Duhok, College of Medicine, Department of General Surgery, Duhok, Kurdistan Region, Iraq; b University of Sulaimani, College of Medicine, Department of General Surgery, Sulaimani, Kurdistan Region, Iraq; c Kscien Organization, Hamdi Str. Azadi Mall, Sulaimani, Kurdistan Region, Iraq

**Keywords:** Intragastric balloon, Deflation, Migration, Bowel obstruction

## Abstract

Intestinal obstruction resulted from balloon migration is an extremely rare but serious late complication of the intragastric balloon (IGB). The aim of this study is to report a case of small bowel obstruction occurring in a middle age corpulent female following embedding of IGB. A 47-year-old obese female presented with abdominal pain, nausea, and vomiting for two days. She had a history of an endoscopically placed IGB nine months before presentation. Physical examination showed an obese woman with mild distress, and the right upper abdomen was tender. The plain abdominal radiograph showed gas shadow in the stomach and the duodenum, esophago-gastro-duodenoscopy showed an empty stomach and balloon migration from the stomach. Under general anesthesia, laparotomy was performed, a three-centimeter antimesenteric enterotomy was done and the balloon extracted from the proximal jejunum. Intestinal obstruction is an extremely rare complication of IGB. It should be managed by laparotomy and extraction of the balloon.

## Highlights

• Intestinal obstruction resulted from intragastric balloon migration is an extremely rare complication.
• It is serious complication and needs general anesthesia and laparotomy.
• In this study, a case of small bowel obstruction occurring in a middle age female following inserting of intragastric ballon has been presented and discussed.

## 1 Introduction

Although their clinical outcome has not been confirmed, for the last two decades, insertion of IGB has been increasingly practiced in the management of obesity. Some studies demonstrated IGB superiority in comparison with diet regimen [1]. Up to date, three types of IGB have been produced; Bioenteric intragastric balloon (BIB) which is filled with liquid was the first to be commercially available. Heliosphere Bag and Endogast were the other two types which were filled with air, the latter necessitates combined endoscopy and surgical approaches to be inserted [2]. Recently, IGB has been proposed to replace bypass operation for the management of obesity [3,4]. Several complications have been reported. Early negative outcomes include nausea and epigastric pain developing a few hours after the procedure leading to balloon extraction in 4.2% [1,2]. Intermediate and late complications include but not limit to gastro-esophageal reflex, gastric ulcers, gastric perforation, and death [4].

Intestinal obstruction resulted from balloon migration is an extremely rare but serious late complication of IGB occurring in 0.8% of the cases [5]. The aim of this study is to present and discuss small bowel obstruction occurring in a middle age corpulent female following embedding of IGB. The work has been reported in line with SCARE guideline [6].

## 2 Presentation of case

**Fig. 1:**
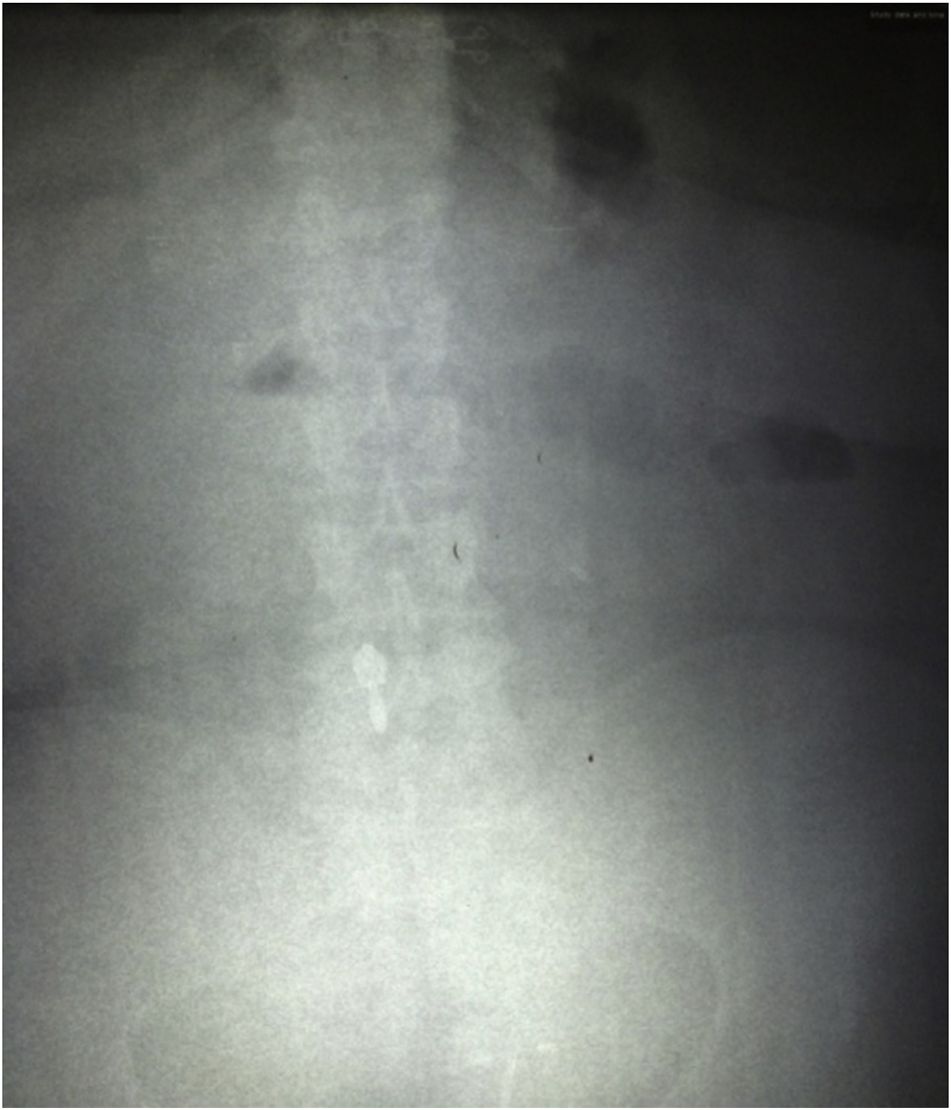
Upright abdominal radiograph revealed gas shadow in the stomach and duodenum.

**Fig. 2:**
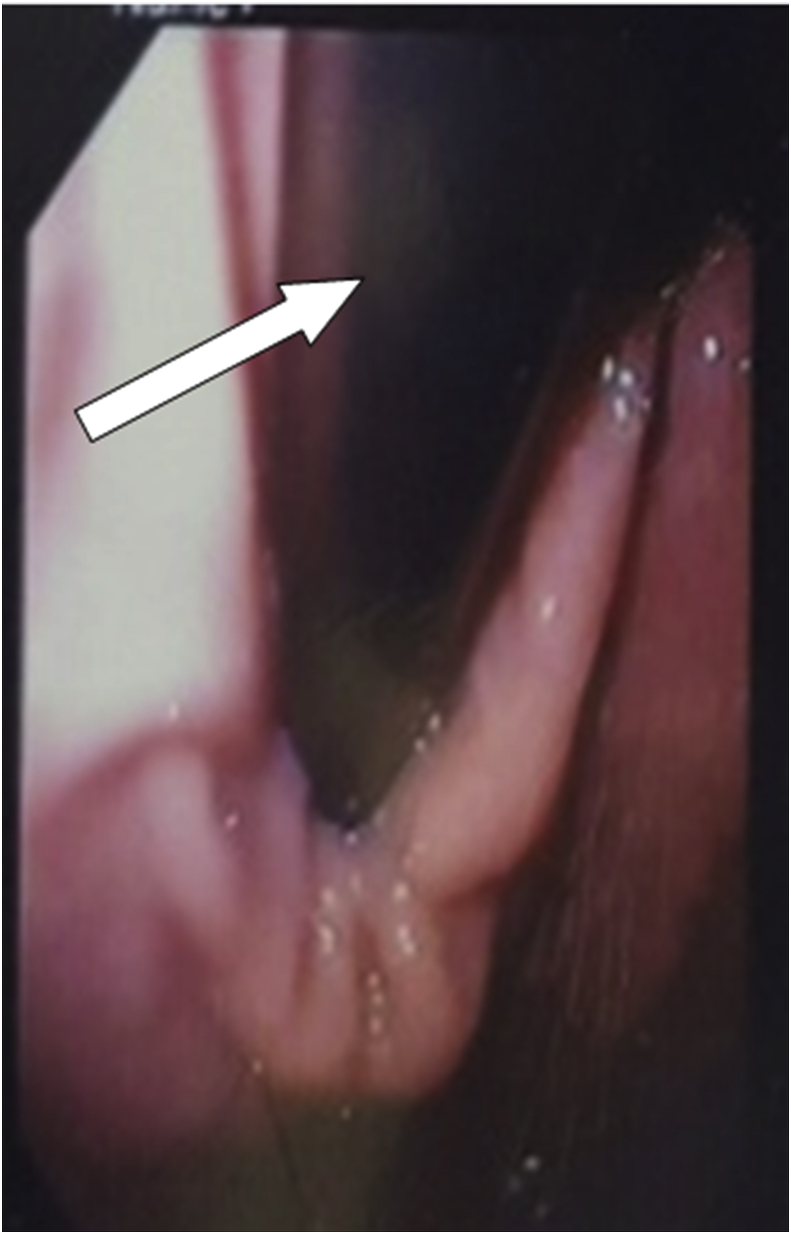
Esophago-gastro-duodenoscopy showing duodenum with balloon migration (white arrow).

**Fig. 3:**
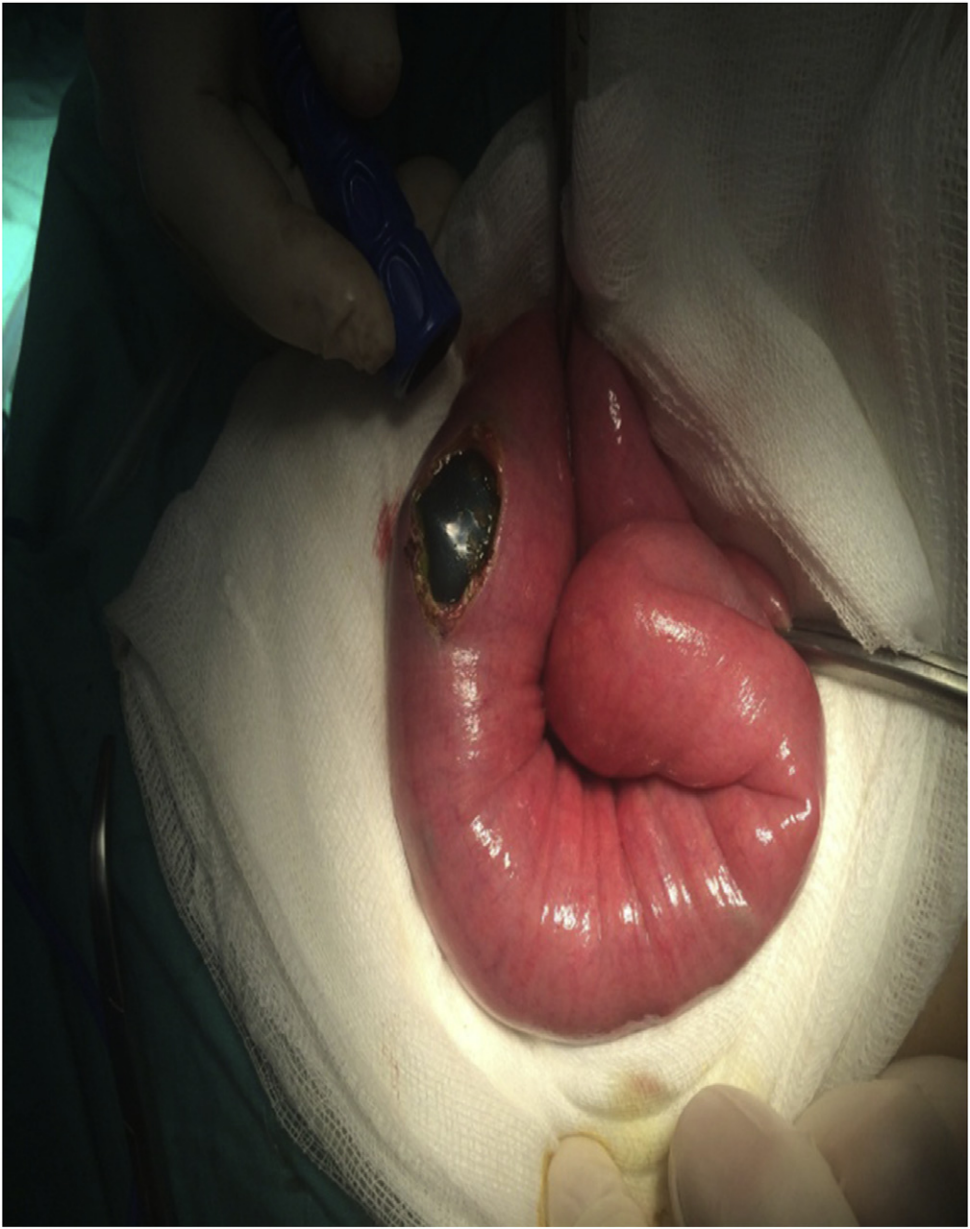
Intraoperative findings showing antimesentericenterotomy with balloon bulging from the bowel lumen.

**Fig. 4:**
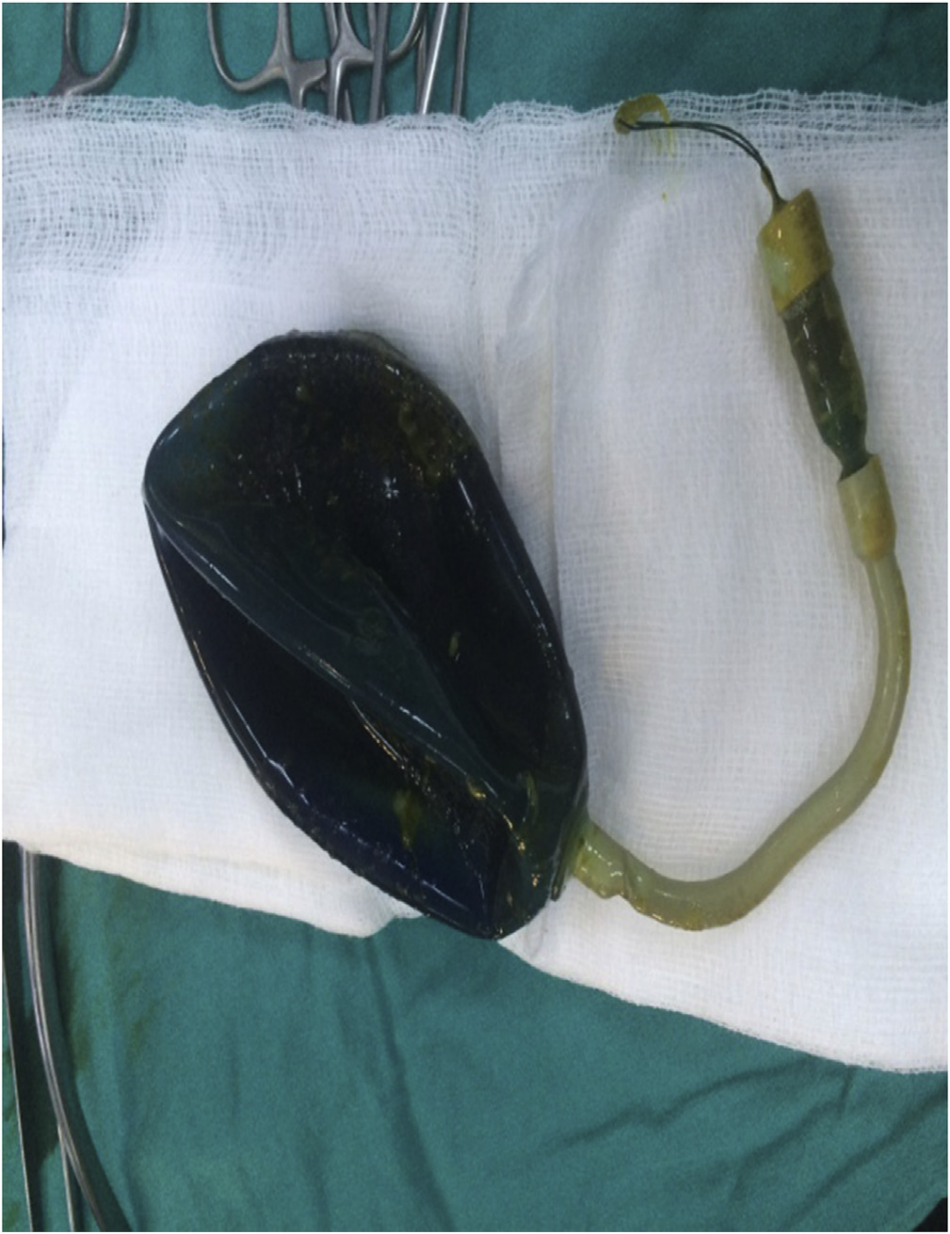
The intragastric balloon after it had been extracted from the jejunal lumen.

A 47-year-old obese female (body mass index (BMI) 37 kg/m^2^) presented to the emergency department with cramp-like abdominal pain, attacks of nausea and vomiting for two days. The pain was in the central region of the abdomen, not responding to analgesics but relieved by vomiting. The patient reported having an endoscopically placed IGB (Spatz3 Adjustable Balloon system^®^, USA) nine months before the presentation. The total weight loss was 15 kg (BMI was 41 kg/m^2^ at placement). She had negative past medical, past surgical and drug history. Physical examination showed an obese woman with mild distress, the abdomen was soft and did not distend; there was tenderness in the upper abdomen without guarding or rebound. Her bowel sound was positive. Blood pressure; 130/80 mmHg, pulse rate; 78 bpm, respiratory rate 15 bpm, temperature 37.3 C, Hemoglobin; 13 g/dl, WBC 7 × 10^9^/L. The plain abdominal radiograph showed gas shadow in the stomach and the duodenum with no air-fluid levels (Fig. 1), Esophago-gastro-duodenoscopy showed an empty stomach and balloon migration from the stomach (Fig. 2). The patient admitted to the surgical ward for two days, correction of hydration status and electrolytes was done. After giving single intravenous antibiotics (ceftriaxone vial 1 g), laparotomy was performed by the first and the second authors in which the bowel was explored. The stomach and proximal jejunum dilated and the palpable balloon was observed in proximal jejunum 40 cm from the duodenojejunal junction, no sign of bowel ischemia or perforation was found. A three-centimeter antimesenteric enterotomy was done (Fig. 3) and the balloon extracted from the jejunum (Fig. 4), the enterotomy closed transversely in two layers. The patient stayed in the hospital for two days and discharged well. The patient was healthy two months later.

## 3 Discussion

Therapy with IGB has been used for decades as a minimally invasive approach to treat obesity despite the inconsistent evidence regarding its efficacy [1,2]. In the current case, the patient satisfied and lost about 15 kg.

The clinical use of an inflatable IGB was reported for the first time in 1982 in a series of five women [3]. The bezoar stayed inflated for about one to three weeks and appeared to decrease weight by reducing hunger. A longer lasting air-filled IGB was invented in the mid-1980 [4]. A gastric balloon with fluid content was invented in the late 1990's and it was quickly accepted a treatment of choice for obesity, replacing its predecessors [5]. It was placed endoscopically as a temporary option accelerating weight loss by filling the stomach which induced satiety feeling and assisted in providing proper dietary customs. The placement of the previous generations of balloons was limited to six months [5]. The Spatz Adjustable balloon (SAB) was invented in 2010 which was the first one to be implanted for one year with adjustability task that changes the balloon volume as preferred. It was approved for patients with BMI >27 kg/m^2^ with failed previous attempts at weight loss [7,8]. The later was put for the current case.

Therapy with IGB, regardless of the types of device, is associated with transient and minor postoperative discomfort (30%). Less commonly, it is associated with major complications such as gastric perforation (0.1%), gastric ulcers (0.5%) and spontaneous deflation (3–23%) with potentially subsequent small-bowel obstruction (0.8%) [1] [2].

Intestinal obstruction subsequent to a spontaneous emptying of an IGB has been reported to occur within a few months of placement [7,8]. In the present case, jejunal obstruction occurred nine months after the procedure.

If IGBs remain longer than the scheduled period, it may spontaneously deflate, migrate and result in bowel obstruction. Most patients have been managed by an operation; however, double-balloon enteroscopy appears a promising alternative to treat intestinal obstruction induced by partially deflated IGBs [[9], [10], [11], [12], [13]].

The learning point in this study is that, in any patient after IGB insertion, close observation and awareness about the serious complications like intestinal obstruction is mandatory. Early diagnosis of rupture and migration may be treated by endoscopy but intestinal obstruction should be managed by laparotomy and extraction of the balloon from the bowel lumen.

## Informed consent

A written informed consent has been taken from the patient for the publication of this report.

## Ethical approval

Approval has been taken from Kscien organization for scientific research, no. 10.

## Sources of funding

No source to be stated.

## Author contribution

**Ayad Ahmad Mohammed**: The team leader, revising the draft with final approval of the manuscript.

**Sardar Hasan Arif**: Revising the draft, reviewing the literature and follow up with final approval of the manuscript.

**Abdulwahid M. Salih**: The surgeon who did the first operation. Final approval of the manuscript.

**Fahmi Hussein Kakamad**: Writing the manuscript, reviewing the literature and final approval of the manuscript.

## Conflicts of interest

There is no conflict to be declared.

## Research registration number

Researchregistry1412.

## Guarantor

Fahmi Hussein kakamad.

## Appendix A Supplementary data

check list check list
